# Harmonizing efficacy and safety: the potentials of CAR-NK in effectively addressing severe toxicities of CAR-T therapy in mantle cell lymphoma

**DOI:** 10.1097/JS9.0000000000001638

**Published:** 2024-05-27

**Authors:** Xu Sun, Yijun Wu, He Li, Ailin Zhao, Ting Niu

**Affiliations:** a Department of Hematology, West China Hospital, Sichuan University; b Division of Thoracic Tumor Multimodality Treatment, Cancer Center, West China Hospital, Sichuan University; c Laboratory of Clinical Cell Therapy, West China Hospital, Sichuan University, Chengdu, Sichuan, People’s Republic of China

*Dear Editor,*

We recently read with interest a critical appraisal published in *International Journal of Surgery* that focused on advances in chimeric antigen receptor T-cell (CAR-T) therapy for patients with mantle cell lymphoma (MCL)^1^. MCL is a subtype of non-Hodgkin B-cell lymphoma, typically characterized by the translocation of chromosome t(11;14)(q13;q32) associated with the overexpression of Cyclin D1^2^. Despite exhibiting features of indolent lymphomas, MCL also demonstrates aggressive behavior, with a limited response to chemotherapy and a high risk of recurrence, leading to a worse survival outcome^3,4^. While the advent of Bruton’s tyrosine kinase inhibitors has somewhat improved the prognosis of relapsed or refractory mantle cell lymphoma (R/R MCL) patients, the clinical challenges still remain and are highly required to be addressed^5,6^. Besides, other conventional therapies like allogeneic hematopoietic stem cell transplantation are limited with a high mortality, thus serving as a treatment option for only a small fraction of patients^7^.

CAR-T therapy has emerged as one of the most rapidly evolving adoptive cell therapies in recent years, achieving landmark success in the field of hematologic malignancies^8^. Currently, there are a total of 11 CAR-T products approved for clinical treatment globally. In April 2020, a multicenter phase 2 clinical trial (ZUMA-2) published in the *New England Journal of Medicine* evaluated the efficacy of KTE-X19 (brexucabtagene autoleucel, a CD19-targeted CAR-T with CD28 co-stimulatory domain) in R/R MCL^9^. In the primary efficacy assessment that enrolled 60 patients, 93% (95% CI: 84–98%) of them had an objective response (OR) and 67% (95% CI: 53–78%) achieved a complete response (CR). After a median follow-up period of 12.3 months (ranging from 7.0 to 32.3 months), a remission was observed in 57% of them. However, despite the encouraging efficacy, safety concerns remain to be noted significantly. It was reported that 99% of subjects experienced ≥ 3-grade adverse events (AEs), particularly hematologic toxicities (cytopenias, 94%). The cytokine release syndrome (CRS) of grade 3 or higher was experienced in 15% of patients, while neurologic events of similar severity were observed in 31% of patients. Two patients (3%) who underwent pre-treatment chemotherapy experienced grade 5 AEs. In another clinical trial, TRANSCEND involving MCL patients, CAR-T therapy showed similarly impressive efficacy (OR: 84%, CR: 59%) with improved safety, yet still with 34% of patients experiencing grade 3 or higher AEs, especially hematologic toxicity-related events^10^. A retrospective study of 26 R/R MCL patients found that 17 patients (65%) experienced immune effector cell-associated neurotoxicity syndrome (ICANS) after the treatment with brexucabtagene autoleucel, 38% (*N*=10) of which were grade ≥3 events, and all of them also experienced a CRS beforehand. Therefore, although CAR-T therapy has become a highly promising option for MCL, treatment failures due to adverse reactions continue to greatly limit the application of CAR-T therapy.

To overcome the toxicity issues associated with CAR-T therapy, the concept of developing CAR-modified natural killer (CAR-NK) cells as iterative products has been proposed in recent years^11,12^. NK cells are innate immune effector cells with direct cytotoxic functions, capable of recognizing malignant cells with a broad specificity without antigen presentation by dendritic cells, thus independent of MHC restriction^13,14^. CAR-NK cells not only possess efficient killing and recognition capabilities but also offer higher practicality and safety than CAR-T: (1) Off-the-shelf potential for universal use: since NK cells do not rely on MHC for antigen recognition, allogeneic NK cells from the same species do not cause graft-versus-host disease (GVHD), making CAR-NK potentially usable as universal cell therapy products for pre-production, which can significantly reduce patient treatment wait times and production costs^15^. (2) Improved safety: CRS is one of the major AEs of CAR-T therapy, induced by pro-inflammatory factors like IL-2, TNF-α, IL-1, and IL-6 secreted by T cells, while NK cells typically secrete IFN-γ and GM-CSF (granulocyte-macrophage colony-stimulating factor), potentially avoiding severe CRS during the treatment^16,17^. (3) Better efficacy: Due to the natural cytotoxic activity of NK cells, in addition to CAR-dependent killing approach, CAR-NK cells can also clear tumor cells through mechanisms like antibody-dependent cellular cytotoxicity (ADCC), exerting stronger killing effects and effectively clearing heterogeneous tumor cells that do not express CAR-targeted antigens^18,19^. Nowadays, a phase I clinical trial including R/R MCL patients is currently evaluating the safety and tolerability of allogeneic CAR-NK cell therapy targeting CD19 (NCT05020678). Therefore, we believe that considering the toxicity limitation of CAR-T therapy for MCL, CAR-NK cells, with great potential for both efficacy and safety, maybe a more promising alternative for CAR-T in the future.

## Ethics approval

Not applicable.

## Consent

Not applicable.

## Sources of funding

This work was supported by Postdoctoral Fellowship Program of CPSF (No. GZB20230481), National Natural Science Foundation of China (No. 82303773, 82303694, 82370192), Postdoctor Research Fund of West China Hospital, Sichuan University (No. 2024HXBH149), 1.3.5 Project for Disciplines of Excellence, West China Hospital, Sichuan University (No. ZYJC21007), Key Research and Development Program of Sichuan Province (No. 2023YFS0031), National Key Research and Development Program of China (No. 2022YFC2502600, 2022YFC2502603).

## Author contribution

X.S., Y.W. and H.L.: writing the paper; A.Z. and T.N.: study concept and review and editing.

## Conflicts of interest disclosure

There are no conflicts of interest.

## Research registration unique identifying number (UIN)

Not applicable.

## Guarantor

Prof Ailin Zhao.

## Data availability statement

Not applicable.

## Provenance and peer review

Not applicable.
